# Supplementary material for: Does Hemoperfusion Increase Survival in Acute Paraquat Poisoning? A Retrospective Multicenter Study
Source: Toxics. 2020 Oct 10;8(4):84. doi: 10.3390/toxics8040084 (PMC7711471; doi:10.3390/toxics8040084)
Supplement: Supplementary file 1 [file toxics-08-00084-s001.pdf]

Supplementary Materials: Does Hemoperfusion Increase Survival in Acute Paraquat Poisoning? A Retrospective Multicenter Study

Ying-Tse Yeh, Chun-Kuei Chen, Chih-Chuan Lin, Chia-Ming Chang, Kai-Ping Lan, Chorng-Kuang How, Hung-Tsang Yen, and Yen-Chia Chen

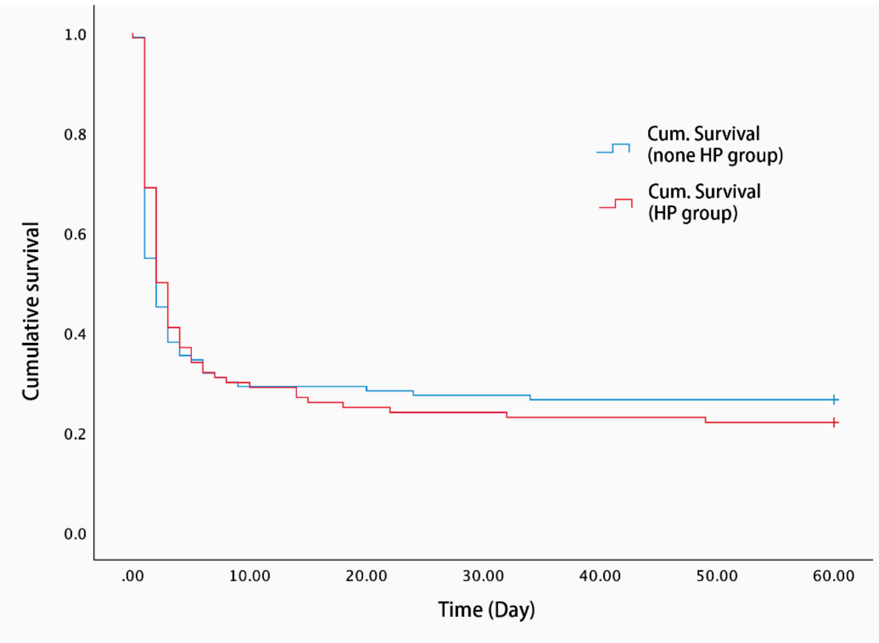

Figure S1. Kaplan–Meier survival analysis between the hemoperfusion (HP) group and non-HP group (p = 0.943, log-rank test).
